# Micronutrient levels and haemato-biochemical status of patients with sickle cell anaemia at a tertiary hospital in Abakaliki, south-eastern Nigeria: a cross-sectional study

**DOI:** 10.11604/pamj.2022.41.303.31728

**Published:** 2022-04-14

**Authors:** Oluomachi Charity Nnachi, Michael Chinwe Orih, Oghenevwogaga Obukohwo Edenya, Augustine Ejike Okoye, Innocent Paul Ezenwenyi

**Affiliations:** 1 Department of Haematology, Alex Ekwueme Federal University Teaching Hospital, Abakaliki, Ebonyi State, Nigeria,; 2 Department of Chemical Pathology, Abia State University Uturu, Abia State, Nigeria,; 3 Department of Chemical Pathology, Alex Ekwueme Federal University Teaching Hospital, Abakaliki, Ebonyi State, Nigeria

**Keywords:** Sickle cell anaemia, micronutrients, renal function, liver function

## Abstract

**Introduction:**

nutritional status is an under-studied environmental factor that can impact the phenotypic manifestations of patients with Sickle Cell Anaemia (SCA). This study aimed to define hemato-biochemical parameters and micronutrient status in patients with SCA.

**Methods:**

this was a cross-sectional study of patients with SCA and hemoglobin genotype HBAA controls at a tertiary health facility in Abakaliki, from 2^nd^ December 2020 to 31^st^ March 2021. Plasma micronutrient levels, haemato-biochemical parameters were analyzed and anthropometric measurements obtained from all participants.

**Results:**

sixty participants with SCA had 58.3% females (mean age of 24.77±7.39 years) while controls had 50% females (mean age of 26.23 ± 8.44 years). The SCA group had significantly lowered calcium (2.733 ± 1.593 vs 1.846 ± 1.123 mmol/l; p=0.009) and magnesium (19.38 ± 6.37 vs 9.65 ± 1.38 mg/dl; p= < 0.001) levels but higher plasma iron (1.70 ± 0.89 vs 1.06 ± 0.53; p=0.001). Zinc and Copper did not reveal significant differences between the two groups. Chloride ion levels was significantly lower in the SCA patients (107.50 ± 17.42 vs 100.19 ± 12.92; p=0.026) while Alkaline phosphatase (ALP), bilirubin, total white blood cell (WBC) and platelets (PLT) count were higher compared with the HBAA group (255.72 ± 124.52 vs 134.56 ± 39.67; p= <0.001, 46.86 ± 25.03 vs 25.63 ± 18.80; p = 0.001, 13.21± 6.57 vs 6.10 ± 1.35; p= < 0.001 and 369.25 ± 138.11 vs 209.36 ± 47.85; p= <0.001).

**Conclusion:**

Copper and zinc deficiency was not present in our population of SCA patients but, they had lower plasma calcium and magnesium levels and elevated levels of blilirubin, ALP, WBC and platelets PLT counts. These parameters can be explored in designing better management for patients with SCA

## Introduction

Sickle cell disease (SCD) is a heritable blood disorder of public health concern in sub-Saharan Africa. The homozygous and most severe form of the disease is known as sickle cell anaemia (SCA). It is characterized by hemolysis, anaemia, recurrent vaso-occlusion, painful crises, inflammation, and progressive multiple end-organ dysfunctions [1]. These conditions increase erythrocyte reactive oxygen species levels with resultant increase in nutrient and energy demand [2]. Reliable data have implicated nutritional disturbances in the pathophysiology of some complications of SCA [3]. Dietary information about SCA is still evolving in Nigeria, and research on macro and micronutrients in SCD is scarce. Micronutrients are vitamins and minerals required by the body in small quantities for cellular metabolic processes. Increased metabolic demand, compensatory diversion, poor appetite, inadequate intake and absorption of micronutrients could exacerbate the burden of sickle cell disease. Magnesium modulates the movement of ions across cellular membranes and is crucial in erythrocyte volume control, which prevents dehydration and destruction in sickle cell pathology [4]. Calcium is critical in red cell membrane stability, deformability, redox state and volume regulation [5]. It is also important in bone formation and mineralization. Iron is important in hemoglobin synthesis, but hyperferritinemia and iron overload are undesired effects. Repeated red cell transfusion, and increased intestinal absorption in SCA, may predispose to iron overload [6].

Copper is essential to the proper functioning of different metalloenzymes involved in iron metabolism [7]. Zinc has antioxidant functions and is involved in growth and development, immune function, cellular metabolism, wound healing, DNA synthesis, and cell division [8]. Severely low haematocrit, high platelet and white cell counts have been implicated in worsening SCA crises and complications, which is associated with disease severity. Liver dysfunction may be due to sequestration of irreversibly sickled red cells, iron deposits and bilirubin gallstones [9]. Renal medullary hypoxia and ischaemia drive renal injury, which may progress to chronic and end-stage renal disease [10]. Data on status of micronutrients in patients with SCA in our locality are scanty, and global data on this subject are meager and conflicting. The assessment of liver function, renal function and complete blood count in patients with SCA is not routine in most of the Nigerian hospitals, hence reported data are few. This study was carried out to evaluate the plasma levels of zinc, copper, iron, magnesium, and calcium, provide baseline data, and define the pattern of hematological, liver, and renal function tests in a population of patients with SCA in our locality.

## Methods

**Study design and setting:** this was a cross-sectional comparative study done at the Sickle Cell Center of Alex Ekwueme Federal University Teaching Hospital (AEFUTH) Abakaliki, Ebonyi state, Nigeria, from 2^nd^ December 2020 to 31^st^ March 2021 2^nd^ December 2020 to 31^st^ March 2021. AEFUTH is a 700- bed capacity government owned tertiary health facility hosting the southeast geopolitical sickle cell center established by the federal government in year 2012.

**Study population:** a total of 120 adult subjects were consecutively enrolled for the study. This consisted of 60 hemoglobin SS homozygous phenotype (SCA) patients attending the sickle cell clinic and 60 control subjects recruited from healthy Haemoglobin AA (confirmed with alkaline medium Hb electrophoresis) blood donors from blood donor clinic.

**Inclusion criteria:** adult patients with Sickle Cell Anaemia (SCA) patients in steady or stable condition.

**Exclusion criteria:** all subjects who were tested positive for Hepatitis B surface antigen, Hepatitis C, HIV/AIDS, history of blood transfusion in the last 3 months, on Zinc, iron, calcium medications and those who did not give consent for the study.

### Data collection

Patient case notes and structured interviewer-administered questionnaires were used to collect demographic and clinical information from the participants. Anthropometric measurement (weight and height) to calculate body mass index (BMI) was done using a weighing scale and stadiometer. BMI was calculated using the formula; BMI = Weight (Kg)/height (m^2^). The World Health Organization classification of BMI was used to categorize our study participants. Under strict asepsis, 10mls of venous blood was collected using a syringe with a 19G hypodermic needle. Two and a half milliliters (ml) and 8mls aliquots were immediately transferred into sodium ethylenediaminetetraacetic acid (EDTA) and 2 lithium heparin sample tubes (4ml each) respectively. The heparin samples were sent to the laboratory for separation by centrifugation at 4000 radians per minute for 10 minutes. One of the plasma aliquots was used to analyze the biochemical parameters the same day, while the second plasma aliquot was stored at -20oc for micronutrients analysis in two batches.

### Laboratory analysis

Biochemical parameters; Alanine Transaminase (ALT), Aspartate Transaminase (AST), Alkaline Phosphatase (ALP), Bilirubin, urea, and creatinine were done with SELECTRA PROx chemistry analyzer using ELITECH kit while serum electrolytes (k+, Na+, Cl- and HCO3-) assay was performed by Sensa Core 200cc electrolyte analyzer. Plasma levels of the micronutrients zinc, iron, calcium, copper, and magnesium were measured with atomic absorption spectrometer (FS240AA Agilent technologies the USA), using the direct air acetylene flame method. Quality control and assurance techniques were used to improve the precision and accuracy of the analytical instrument. The blanks were used to check contamination during sample preparation. Measurements were done in duplicates to check the reproducibility of the method used. The standards used were to check the efficiency of the equipment used. Complete blood count and red cell indices of the Na EDTA samples were measured using an automated haematology analyzer (Mindray BC 5300). Haematological and biochemical tests were done at the Research laboratory of AEFUTHA. The duration of the study was 5 months.

**Statistical analysis:** analysis of data was done using IBM SPSS Statistics for windows, version 19.0. Amork, NY: IBM Corp (2010). Results were expressed as mean ±SD. Pearson chi-square and Fisher's exact test were employed for data analysis, a P = 0.05 was considered to be statistically significant.

**Ethical considerations:** the study was approved by the Research and Ethics Committee of the Hospital (REC PROTOCOL NUMBER 17/12/2020-10/02/2021). Written informed consent was obtained from the participants.

## Results

### Sociodemographic characteristics of the study population

The study participants with SCA had a mean age of 24.77 ± 7.39 years and more than half (58.3%) were females, while the control group had a mean age of 26.23 ± 8.44 years with equal population of both sex. There was a statistically significant difference between the mean height (1.64 ± 0.09 vs 1.72 ± 0.08 m; p =< 0.001), weight (56.25 ± 11.50 vs 74.00 ± 12.86 kg; p= < 0.001), and Body Mass Index (BMI) (20.69 ± 3.36 vs 24.85 ± 4.30; p =< 0.001) of the study groups (Table 1).

**Table 1:** socio-demographic characteristics of the study population

	SCA (n=60)	Control (n=60)	
Parameter	Mean (SD)	Mean (SD)	P-value
Age (years)	24.77(7.39)	26.23(8.44)	0.072
Gender			
Male	25 (41.7)	30 (50)	0.460
Female	35 (58.3)	30 (50)	
Height (m2)	1.64(0.09)	1.72(0.08)	<0.001
Weight (kg)	56.25(11.50)	74.00(12.86)	<0.001
BMI (kg/m2)	20.69(3.69)	24.85(4.30)	<0.001

Large difference between the BMI of the study population which is highly significant (t = - 6.62; p = < 0.001)

### Micronutrient profile of the study population

The mean level of plasma iron was 1.70 ± 0.89 mg/dl in the SCA study population, versus 1.06 ± 0.53 mg/dl in the control group (p < 0.001). The plasma level of calcium (1.846 ± 1.123 vs 2.733 ± 1.593 mmol/l; p = < 0.05) and magnesium (9.65 ± 1.38 vs 19.38 ± 6.37 mg/dl; p = < 0.05) were significantly lower in the SCA group compared with control group while Copper (0.55 ± 0.35 vs 0.58 ± 0.28 mg/dl; p=0.699) and Zinc (0.79± 0.31 mg/dl vs 0.79 ± 0.38 mg/dl; p=0.935) showed no statistically significant difference (Table 2).

**Table 2:** micronutrient levels of the study participants

	SCA (n=60)	Control (n=30)	
Parameter	Mean (SD)	Mean (SD)	P-value
Iron (mg/dl)	1.70(0.89)	1.06(0.53)	0.001
Copper (mg/dl)	0.55(0.35)	0.58(0.28)	0.699
Zinc (mg/dl)	0.79(0.31)	0.79(0.38)	0.935
Calcium (mmol/l)	1.846(1.123)	2.733(1.593)	0.009
Magnesium (mg/dl)	9.65(1.38)	19.38(6.37)	<0.001

* Significant at p <0.05 - Iron and magnesium

### Biochemical profile of study population

In the Biochemical profile of the study population, the levels of ALP (255.72 ± 124.52 vs 134.56±39.67; p= < 0.001) total bilirubin (46.86 ± 25.03 vs 25.63 ± 18.80; p= 0.001) and conjugated bilirubin (23.09 ± 26.24 vs 8.92 ± 7.00; p= 0.008) were significantly higher in SCA patients compared with the control group. Apart from the level of Chloride ion that was significantly lower in the SCA patients (100.19 ± 12.92 vs 107.50 ± 17.42; p=0.026), other electrolytes (sodium, bicarbonate, and potassium), creatinine, and urea were not significantly different from the HbAA group (Table 3).

**Table 3:** biochemical profile of study participants

	SCA (n=60)	Control (n=30)	
Parameter	Mean (SD)	Mean (SD)	P-value
Urea	6.74(23.32)	1.88(0.97)	0.259
Creatinine	72.79(80.39)	74.96(28.46)	0.886
Sodium	136.24(18.57)	138.93(3.13)	0.434
Potassium	4.99(8.42)	5.19(8.95)	0.536
Chloride	100.19(12.92)	107.50(17.42)	0.026
Bicarbonate	23.26(3.08)	24.22(1.82)	0.122
Total bilirubin	46.86(25.03)	25.63(18.80)	0.001
Conjugated bilirubin	23.09(26.24)	8.92(7.00)	0.008
ALT	47.96(91.67)	9.84(6.72)	0.025
AST	49.36(58.58)	21.52(9.02)	0.009
ALP	255.72(124.52)	134.56(39.67)	<0.001

Significant p< 0.05- Chloride, Total bilirubin, Alanine transaminase-AT, and Alkaline phosphatase-ALP

### Hematological variables

The total WBC was markedly different between the SCA group (13.21±6.57) and the HbAA group (6.10 ± 1.35; p = < 0.001). The white blood cell (WBC) differentials were also significantly different between the groups. As might be expected, the hemoglobin concentration (7.85 ± 1.64g/dl vs 12.73±1.82g/dl) and RBC count (2.97±0.74 vs 4.49 ± 0.65) were significantly lower in the HbSS patients. The Mean Corpuscular Volume (MCV) was significantly lower (81.90 ± 9.02 vs 86.80 ± 7.87; p=0.013) while platelet count was higher (369.25 ± 138.11 vs 209.36±47.85) in the SCA group compared to the HBAA controls (Table 4).

**Table 4:** hematological variables of the study participants

	SCA (n=60)	Control (n=30)	
Parameter	Mean (SD)	Mean (SD)	P-value
Total WBC	13.21(6.57)	6.10(1.35)	<0.001
Absolute NC	8.34(7.69)	2.78(0.91)	<0.001
Absolute LC	4.48(2.45)	2.82(0.66)	<0.001
HCT	23.77(4.44)	36.96(5.93)	<0.001
Hgb	7.85(1.64)	12.73(1.82)	<0.001
RBC	2.97(0.74)	4.49(0.65)	<0.001
MCV	81.90(9.02)	86.80(7.87)	0.013
MCH	27.89(3.56)	28.52(3.10)	0.412
MCHC	38.60(37.51)	32.81(1.19)	0.402
Platelet count	369.25(138.11)	209.36(47.85)	<0.001

WBC - White blood cell count, NC- Neutrophil count, LC- Lymphocyte count, HCT- Haematocrit, Hgb -Haemoglobin, RBC - Red blood cell, MCV - Mean corpuscular volume, MCH - Mean corpuscular haemoglobin, MCHC - Mean corpuscular haemoglobin concentration

## Discussion

The pathophysiology of SCA increase erythrocyte reactive oxygen species level and cell turnover which results in increased nutrient and energy demand which can exacerbate disease burden [1]. Body Mass Index is a measure for indicating nutritional status in adults. In the current study, the low BMI of the patients with SCA could be attributable to the increased resting energy expenditure, hypermetabolism, nutrient diversion, and micronutrient deficiencies [11]. The patients with SCA in our study had lower Mg^2+^ and calcium levels, similar to reports by Yousif *et al*. [12] but contrary to Akenami *et al*. [13]. Increase in glomerular filtration due to glomerular ischaemia and suboptimal reabsorption at the tubules leads to increased Mg2+ urinary loss in SCA [14]. Hypomagnesaemia could also result from increased turnover of haemopoeisis, inadequate nutritional intake, poor absorption and hypoxia-associated red blood cell membrane changes in the activity of Na+-Mg2+ exchanger which promotes magnesium efflux. Intracellular hypomagnesaemia activates K^+^-Cl^+^co-transporter which causes red cell dehydration, sickling, vaso-occlusion, and hemolysis in SCA. In agreement with Mohammed *et al*. the current study found low calcium levels in patients with SCA while another study reported normal levels [15, 16]. The disturbance in calcium homeostasis may be due to impaired vitamin D synthesis, altered renal handling, reduced dietary supply, reduced intestinal absorption, and high intra-erythrocyte calcium uptake due to red cell membrane abnormalities. Hypocalcaemia promotes sickling and may contribute to the skeletal changes of the disease. Raised plasma iron levels were observed in our SCA population, similar to the report of Sani *et al*. [17] but contrary to the findings of Ray *et al*. [18]. Increase in body iron may result from transfusion iron load, nutritional, socioeconomic, and/or genetic factors. Iron overload has deleterious effects that can complicate SCA pathology, hence meticulous use of red cell transfusion and regular assessment of the iron status of SCA patients should be incorporated in their management plan.

In agreement with Alayash *et al*. [19] but contrary to Okocha *et al*. and Bot *et al*. [20, 21], our study concluded that the zinc and copper levels in SCA patients and those of controls were similar. These variations could be due to differences in dietary sources, absorption or genetic differences of the study population. In resource-poor settings like Nigeria, micronutrient deficiency exists due to the inability to augment staple food with micronutrient-rich diets like red meat, fish, and poultry. Zinc deficiency is reported to be common in the general population of most low-income countries [22] and this may explain the insignificant difference between the SCA study population and the control. Significant increase in bilirubin and ALP levels in patients with SCA was demonstrated in the current study, similar to reports by a previous study [23]. Hepatic infarction due to sinusoidal obstruction by sickled red cells, intra-hepatic sequestration, cholelithiasis, transfusion-associated iron overload, viral hepatitis, and chronic haemolysis are implicated in SCA hepatopathy [9, 24]. Cholelithiasis and increased repair activity of osteoblasts when there is bone infarction following vaso-occlusion in bone could cause increase in ALP levels. Bone complications in SCA could thus be monitored with ALP, which is a sensitive marker of bone turnover. The constant hemolysis in SCA patients increases bilirubin levels and plasma load of AST, while hepatocyte injury from the various causes in SCA may explain the relatively higher values of ALT and AST. The earliest manifestations of sickle cell nephropathy (isosthenuria and urinary acidification defect) do not usually lead to renal function impairment unless there is an acute crisis. This agrees with our renal findings where the patients with SCA had normal plasma electrolytes, urea, and creatinine levels but low chloride levels. Metta *et al*. [25] report corroborated this finding, but contrastingly, Yusuf *et al*. [26] observed significantly higher electrolytes and creatinine levels in SCA patients. Abnormal renal function in SCA, is more common in later years of life and as such was not notable among our study participants with a mean age of 24.77 years.

As expected, the hemoglobin (Hb) levels of the SCA patients in the current study are low and corresponds to reports by Omoti *et al*. [27]. Well-known causes of low hemoglobin levels in SCA are chronic hemolysis, ineffective erythropoiesis, nutritional disturbances, and reduced erythropoietin production. Even with the low hemoglobin levels, SCA patients maintain normal function and activity with their steady-state level, only requiring blood transfusion during intercurrent illnesses and acute crises resulting in acute fall in Hb. High total WBC, differential, and platelet count demonstrated by a previous study in SCA agree with this current study [28] Asplenia with loss of splenic pool, preferential increase in marginating pool, and increased inflammatory cytokines during vaso-occlusive events increases platelet and WBC counts in SCA which impacts on SCA morbidity and mortality. The mean platelet count of 369 x 109 in our patients was higher than reported by Iheanacho *et al*. [28]. The patients with SCA in the current study did not benefit from the cytoreductive effect of hydroxyurea therapy due to financial constraints [29]. In line with reports by Adeyemo *et al*. [30], the index study revealed slightly higher MCHC and lower MCV and MCH in patients with SCA. Increased MCHC is associated with red cell dehydration, which causes the red cells to become dense and prone to hemolysis. In a steady-state, SCA individuals have reduced hemolysis rate and erythropoiesis hence MCV and MCH may near normal values. Inability to assess intracellular red cell levels of the micronutrients due to financial constraints and quantification of dietary constituent and intake of our study participants are the limitations of this study. A multicenter collaboration with a larger population size could have increased the power of the study to detect significant results.

## Conclusion

Our population of patients with SCA had lower calcium and magnesium levels and no significant difference was observed in the renal function parameters, zinc and copper levels of the patients with SCA and HBAA controls. The patients with SCA also had hyperbilirubinaemia, elevated levels of ALP, WBC and Platelet counts, as well as lower Hb and Hematocrit (HCT). These parameters can be explored in designing better management of individuals with SCA.

### What is known about this topic

- Varying micronutrient deficiencies have been identified in patients with sickle cell disease and this can further complicate the disease severity;
- Worldwide data on micronutrients status in sickle cell anaemia is conflicting.

### What this study adds

- Patients with Sickle cell anaemia in our locality has low plasma calcium and magnesium levels.
- Renal function parameters, zinc and copper levels of patients with SCA were not significantly different from HBAA individuals.
